# Longitudinal trajectories of benefit finding and its associations with emotion regulation, social connectedness, depression, and anxiety among Chinese college students following the removal of COVID-19 restrictions. a multivariate latent growth curve analysis

**DOI:** 10.3389/fpsyt.2026.1940327

**Published:** 2026-10-29

**Authors:** Wenbo Wang, Guangyu Wen, Deran Li, Xiaoxiao Liu, Rongjuan Guo, Dongfang Wu

**Affiliations:** 1 Department of Psychosomatic Medicine, Dongfang Hospital, Beijing University of Chinese Medicine, Beijing, China; 2 Department of Student Affairs, Northeast Agricultural University, Harbin, China; 3 Graduate School, Wenzhou Medical University, Wenzhou, China; 4 The First School of Clinical Medicine, Wenzhou Medical University, Wenzhou, China; 5 Second Department of Encephalopathy, Dongfang Hospital, Beijing University of Chinese Medicine, Beijing, China; 6 School of Laboratory Medicine and Life Science, Wenzhou Medical University, Wenzhou, China

**Keywords:** benefit finding, college students, emotion regulation, latent growth curve modeling, mental health recovery, social connectedness

## Abstract

**Background:**

Benefit finding may be associated with better mental health after the pandemic, but its longitudinal trajectory and its associations with psychosocial resources and mental health symptoms during recovery remain unclear.

**Objective:**

This study examined trajectories of benefit finding among Chinese college students from approximately 3 to 15 months after COVID-19 restrictions were lifted. We also examined its associations with emotion regulation, social connectedness, depressive symptoms, and anxiety symptoms, together with indirect association patterns involving benefit finding.

**Methods:**

A three-wave longitudinal study was conducted among 1,155 Chinese college students. Data were analyzed using parallel-process latent growth curve modeling.

**Results:**

Social connectedness and benefit finding increased linearly over time, whereas depressive and anxiety symptoms decreased. Cognitive reappraisal was positively associated with the benefit finding intercept but negatively associated with its slope, whereas expressive suppression was not significantly associated with either growth factor. Social connectedness showed stronger associations with benefit finding trajectories than the emotion regulation variables. Benefit finding was negatively associated with depressive and anxiety symptoms at both the intercept and slope levels. Indirect association patterns through benefit finding were supported for social connectedness and both depressive and anxiety symptoms at the intercept and slope levels when inference was based on bias-corrected bootstrap confidence intervals.

**Conclusions:**

Benefit finding increased during the post-pandemic recovery period and was associated with lower depressive and anxiety symptoms. Social connectedness showed relatively strong associations with benefit finding trajectories, while cognitive reappraisal was associated with higher initial benefit finding but a slower subsequent increase. The indirect association patterns linked social connectedness with both symptom domains through benefit finding at the intercept and slope levels. These findings highlight psychosocial resources that merit further study in campus mental health research, while the observational design does not establish causal direction or temporal precedence.

## 1 Introduction

The COVID-19 pandemic posed a major global health challenge that affected physical health and psychological well-being (1). Although stringent public health measures were necessary to limit viral transmission, they also disrupted social life and everyday functioning, creating additional strain for vulnerable groups. College students may have been particularly susceptible because the pandemic coincided with a period of identity formation and psychosocial development. Disrupted education, social isolation, and uncertainty about the future were associated with substantial increases in depression, anxiety, and other psychological difficulties among university students worldwide. In China, the proportion of depressive symptoms among university students increased from 23.8% before the pandemic to 41.1% during peak lockdown periods (2). In the United States, anxiety among college students approximately doubled during the same period (3).

The psychological effects of the pandemic on college students persisted beyond the acute crisis. The return to routine after restrictions were eased occurred alongside continuing stressors, including the socioeconomic consequences of the pandemic, concern about renewed outbreaks, and broader uncertainty about the future. Post-restriction evidence indicates that mental health concerns remained substantial. A large serial cross-sectional study across 22 colleges in Guangdong Province, China, reported standardized prevalence rates of 19.9% for depression and 13.5% for anxiety after restrictions were lifted (4). Longitudinal network evidence also indicates that anxiety and depressive symptoms remained closely interconnected during the transition from lockdown to lifted restrictions (5).

These continuing mental health concerns are consistent with the possibility that the pandemic functioned as a prolonged stressor and, for some individuals, a collective traumatic experience. They also underscore the continuing need to identify psychosocial resources associated with recovery among college students (6).

Benefit finding is one such resource that has been examined in the context of stress and adversity (7). It refers to the process of identifying positive meaning or perceived gains from difficult life experiences. Unlike resilience, which emphasizes the maintenance or restoration of functioning after adversity, benefit finding centers on cognitive reorganization and the recognition of positive changes following stressful experiences. Meaning-making and cognitive reappraisal are among the processes that may contribute to benefit finding (8). Reframing stressful experiences such as the COVID-19 pandemic and recognizing personal growth or valued changes may help individuals develop a more coherent understanding of what they have experienced. Benefit finding may also coexist with the accumulation of psychological resources such as gratitude and hope, which can broaden coping options and support adaptive functioning (9). Positive psychological changes, including posttraumatic growth, were also observed during the early months of the pandemic (10).

Despite increasing interest in benefit finding, most COVID-19 research has focused on acute lockdown periods. Its trajectory during post-restriction recovery and its longitudinal associations with psychosocial resources and mental health symptoms remain less well understood, although recent work has linked benefit finding with outcomes such as academic engagement (11). The present study therefore uses multivariate latent growth curve modeling to estimate latent intercepts and slopes. The intercept represents initial status at approximately 3 months after restrictions were lifted, and the slope represents linear change over the subsequent 12 months. This approach offers two advantages. First, unlike cross-sectional studies, which provide information only about associations at a single time point, latent growth curve modeling separates individual differences in starting levels from individual differences in rates of change (12). Second, unlike cross-lagged panel models, which focus on lagged associations between repeated measures (13), latent growth curve modeling focuses on how starting levels and rates of change covary across constructs. Because the lifting of restrictions involved a rapid change in students’ social environments, modeling trajectories over time is more informative than modeling associations at a single time point or across adjacent waves.

The present study is guided by a single theoretical framework that specifies how psychological resources may be associated with benefit finding over time. The meaning-making model provides this framework (8). According to this model, individuals hold a global meaning system that includes beliefs, goals, and a sense of purpose. Stressful events are appraised in relation to this system, and discrepancies between global and situational meaning can prompt efforts to restore coherence. This reconciliation process may involve reappraising the meaning of the stressful event, adjusting existing beliefs or goals, or both. Benefit finding can be understood as one possible positive outcome of this meaning-making process. We use complementary theories only to clarify the types of resources that may facilitate meaning-making. Ecological systems theory helps distinguish individual and interpersonal contexts, while the thriving through relationships model and conservation of resources theory offer supplementary perspectives on how social resources may support meaning-making. These complementary perspectives are not treated as independent causal models in the present analyses.

### 1.1 Longitudinal development of benefit finding

Longitudinal findings on benefit finding vary according to the type of adversity, the timing of assessment, and the population studied. In medical contexts such as breast cancer and HIV/AIDS, studies have reported moderate to high stability in benefit finding over time, suggesting that perceived benefits may become relatively stable once established (14, 15). Patterns following collective trauma appear more heterogeneous. A national longitudinal study of adults in the United States after the September 11 attacks found an initial increase in benefit finding followed by a decline (16). Research after earthquakes has identified groups with persistently high, increasing, decreasing, and persistently low trajectories of posttraumatic growth or benefit finding (17). During the acute phase of COVID-19, Chinese healthcare workers also showed heterogeneous trajectories, with most participants demonstrating increasing posttraumatic growth while other subgroups remained low or declined (18). In the general Chinese population, benefit finding showed little change over a one-month interval, although coping strategies were associated with later benefit finding (19). Nationally representative data from the United States similarly showed small but statistically significant increases in posttraumatic growth during the early months of the pandemic (20).

Understanding the trajectory of benefit finding is relevant both theoretically and practically. Longitudinal change can help distinguish gradual psychological adaptation from a short-lived response to acute stress. It can also identify periods in which benefit finding is stable, increasing, or declining, which may inform the timing of future intervention research. Most COVID-19 studies, however, focused on the acute lockdown phase, leaving post-restriction trajectories less well characterized. Among Chinese college students, benefit finding has been reported at moderate levels and has been associated with anxiety and academic engagement, but cross-sectional or repeated cross-sectional designs provide limited information about within-sample change over time (21). Within the meaning-making framework, benefit finding may increase gradually as individuals continue to integrate pandemic experiences into their broader beliefs and goals (8). A complementary developmental perspective also suggests that positive psychological engagement can unfold gradually through ongoing interaction with the environment (22). On this basis, we proposed the following hypotheses.

H1a: Benefit finding will show a moderate initial level followed by gradual linear change over time.

H1b: Significant interindividual variability will be present in both initial benefit finding levels and rates of change.

### 1.2 Variables associated with benefit finding trajectories

Guided by the meaning-making model, we selected two sets of variables that may be relevant to benefit finding trajectories. Emotion regulation strategies represent internal cognitive and behavioral resources that shape how emotional experiences are processed (23). Social connectedness represents an interpersonal resource related to belonging and support (24). Ecological systems theory provides a complementary rationale for examining variables at both the individual and social levels (25). The meaning-making model nevertheless remains the central framework. In this framework, cognitive and social resources may facilitate the appraisal and reinterpretation processes involved in benefit finding, so we examined their associations with both the initial level and subsequent change in benefit finding.

#### 1.2.1 Emotion regulation strategies as time-invariant variables

Emotion regulation strategies are cognitive and behavioral approaches used to modify emotional experience and expression (26). The present study focuses on two commonly studied strategies. Cognitive reappraisal involves changing the interpretation of a situation in order to alter its emotional significance, whereas expressive suppression involves inhibiting the outward expression of emotion. Within the meaning-making model, cognitive reappraisal can directly contribute to the reinterpretation of stressful experiences and may therefore facilitate meaning-making (8). Expressive suppression may be less conducive to this process because it can limit emotional processing and consume cognitive resources (27).

Emotion regulation strategies are often conceptualized as relatively stable dispositional tendencies that reflect habitual patterns of emotional processing, an interpretation supported by evidence of test-retest stability and dispositional consistency (26). They were therefore modeled as time-invariant variables in the present study. Previous studies have reported positive associations between cognitive reappraisal and benefit finding, whereas expressive suppression has shown negative or nonsignificant associations (28–30). We therefore proposed the following hypotheses.

H2a: Cognitive reappraisal will be positively associated with the intercept of benefit finding.

H2b: Expressive suppression will be negatively associated with the intercept of benefit finding.

H2c: Emotion regulation strategies will be associated with the slope of benefit finding trajectories.

#### 1.2.2 Social connectedness as a dynamic variable

Social connectedness refers to the subjective experience of belonging and meaningful interpersonal connection within a social network (31). The thriving through relationships model provides a useful account of the social context in which meaning-making may occur. Supportive relationships can provide emotional security, validation, and opportunities to discuss and reinterpret adversity (32, 33). Within the meaning-making framework, these relational processes are directly relevant to benefit finding (8). Conservation of resources theory offers a complementary perspective on why social connectedness may be especially relevant during recovery (34). Resources can cluster and facilitate access to additional resources, such as greater emotional support, exposure to alternative perspectives, and opportunities to construct meaning collaboratively.

As described above, social connectedness may provide a relational context for meaning-making and thereby support benefit finding. Prior intervention and cross-lagged studies provide empirical support for examining this direction. Structured interventions designed to enhance social resources or meaning-making have been associated with improvements in benefit finding or related adaptive outcomes in some populations (35), and cross-lagged analyses following the Wenchuan earthquake showed that social support prospectively predicted posttraumatic growth at earlier time points, although the strength of this association varied across periods (36). Importantly, the direction specified in the present models is also supported by the contextual features of the post-restriction period. During lockdowns, face-to-face contact among college students was at a historically low level; after restrictions were lifted, it expanded rapidly. This shift was driven primarily by macro-level policy changes rather than by individual-level cognitive change, which makes social connectedness the more plausible starting point during this particular window. Although the reverse direction cannot be excluded, the theoretical, empirical, and contextual considerations described above provide a rationale for examining this direction.

H3: The intercept and slope of social connectedness will be positively associated with the corresponding intercept and slope of benefit finding. Higher initial social connectedness and greater increases in social connectedness are expected to coincide with higher initial benefit finding and more positive benefit finding trajectories.

### 1.3 Associations of benefit finding with depression and anxiety

The next set of analyses examined how benefit finding trajectories were associated with depressive and anxiety symptoms, two central indicators of post-pandemic adjustment. The meaning-making model suggests that benefit finding may be related to better emotional adjustment because it involves changes in appraisal, meaning, and perceived agency (8). These processes may be relevant to depressive cognitions such as helplessness and negative beliefs about the future, as well as to anxious threat appraisal. Evidence from intervention and longitudinal studies is broadly consistent with these associations. A cluster-randomized trial found that a benefit-finding intervention reduced depressive symptoms among caregivers (37). Longitudinal work among cancer survivors has also linked benefit finding with subsequent well-being and lower emotional distress (38, 39). These studies provide a rationale for examining the corresponding longitudinal associations in college students, although the same mechanisms may not operate in the present sample.

H4a: Higher initial benefit finding and a more positive benefit finding trajectory will be associated with lower initial depressive symptoms and greater reductions in depressive symptoms over time.

H4b: Higher initial benefit finding and a more positive benefit finding trajectory will be associated with lower initial anxiety symptoms and greater reductions in anxiety symptoms over time.

### 1.4 Indirect association models

We also examined whether the pattern of associations among social connectedness, benefit finding, and mental health symptoms was consistent with statistical mediation. The indirect association model follows from the theoretical sequence established above: social connectedness provides a relational context for meaning-making, benefit finding represents one outcome of that process, and successful meaning-making is expected to reduce distress. In the parallel-process latent growth models, the intercept and slope factors of social connectedness were entered as model inputs, the intercept and slope factors of benefit finding as intervening variables, and the intercept and slope factors of depression or anxiety as model outputs, with cognitive reappraisal and expressive suppression initially considered as time-invariant covariates. These specifications test indirect associations among latent growth factors and reflect the theoretical sequence proposed by the meaning-making model rather than an empirically established causal order. The hypotheses are therefore stated in terms of indirect statistical associations.

H5a: An indirect association through the benefit finding intercept will link the social connectedness intercept with the depression and anxiety intercepts.

H5b: An indirect association through the benefit finding slope will link the social connectedness slope with the depression and anxiety slopes.

H5c: An indirect association through the benefit finding slope will link the social connectedness intercept with the depression and anxiety slopes.

The proposed research model is presented in **Figure 1**.

**Figure 1:**
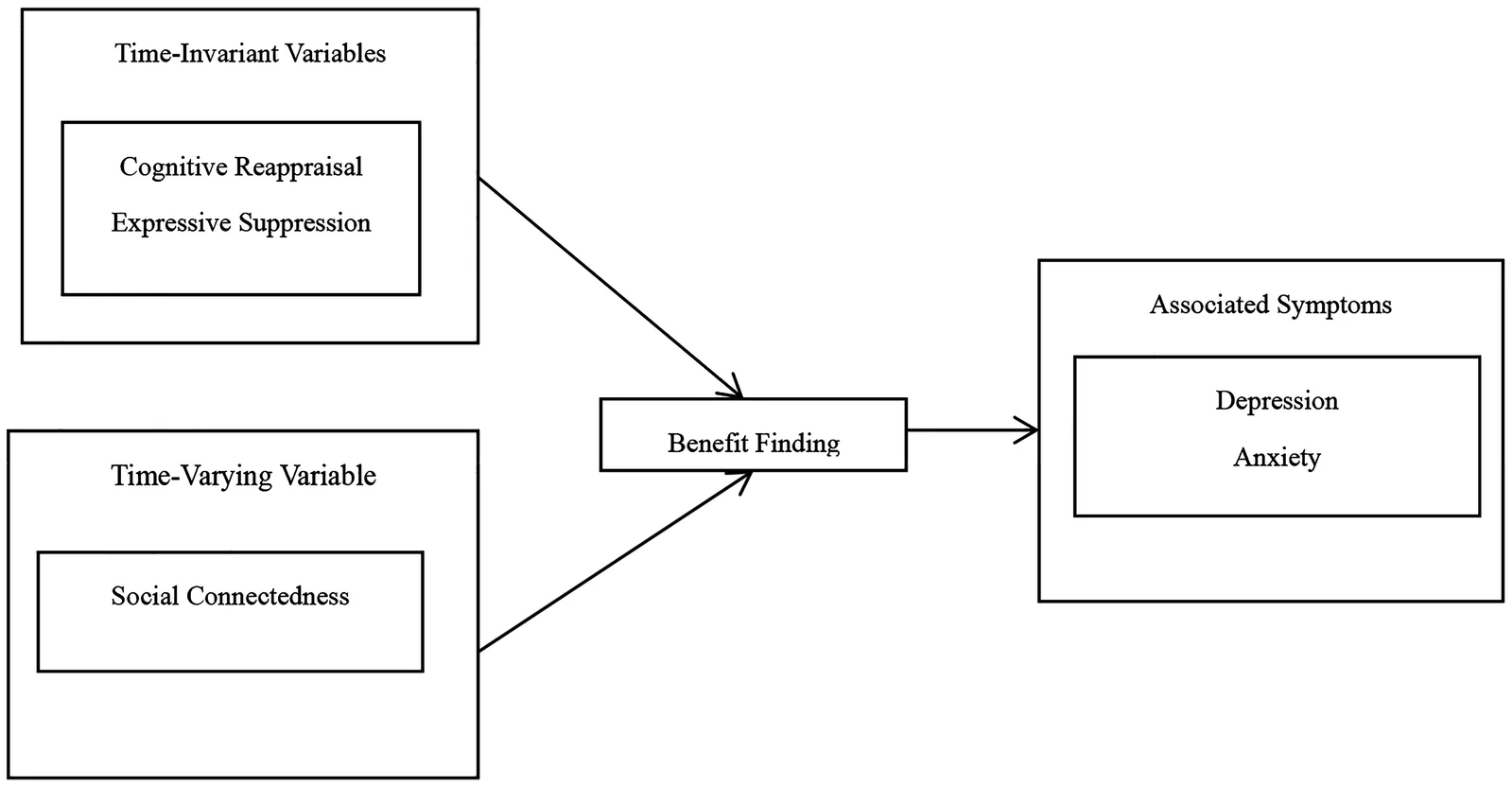
Conceptual model of the hypothesized associations. Arrows indicate theory-specified model paths and do not imply causal effects.

## 2 Materials and methods

### 2.1 Participants and procedure

This study used cluster random sampling to recruit undergraduate students from two universities in Wenzhou, Zhejiang Province, and Harbin, Heilongjiang Province, China. All participants voluntarily completed a comprehensive mental health assessment free of charge. Eligibility criteria were age 18 years or older, current enrollment as an undergraduate student, and no diagnosed psychiatric disorder. After completing the assessment, eligible participants received a detailed mental health report.

The study used a prospective longitudinal design with assessments conducted between April 2023 and April 2024. These time points were selected to represent relatively early, middle, and later stages after the end of COVID-19 preventive restrictions. Time 1 was conducted in April 2023, approximately 3 months after the measures ended. Time 2 was conducted in October 2023, approximately 9 months after the measures ended. Time 3 was completed in April 2024, approximately 15 months after restrictions were removed. Consecutive assessments were therefore separated by approximately 6 months.

At Time 1, 1,181 Chinese college students completed the self-report questionnaires. Of these participants, 62.6% were female, and the mean age was 19.97 years with an SD of 0.96. Participants were invited to complete Time 2 after 6 months and Time 3 after 12 months. Twenty-six participants were unavailable for one or more follow-up assessments or were excluded because of school suspension, off-campus internships, or invalid responses. Invalid responses included incorrect or inconsistent answers to attention-check items such as the arithmetic item asking respondents to calculate 6 plus 3. The final longitudinal sample comprised 1,155 students. At Time 1, 62.2% of this sample was female, and the mean age was 19.97 years with an SD of 0.97. The retention rate was 97.8%.

Data were collected through the Wenjuanxing platform, which participants accessed by scanning a QR code. Data validation procedures were applied before analysis. Records with inconsistent demographic information and responses that failed the attention checks were excluded. Names were not collected for the analytic dataset, although student identification numbers were retained to link responses across waves. Participation was voluntary, and participants could withdraw at any time without penalty. Before opening the questionnaire, each participant was required to read an electronic informed consent statement and indicate agreement to participate by selecting the consent button. Participants who did not provide electronic consent could not proceed to the questionnaire. This electronic consent procedure was approved by the Ethics Committee of Wenzhou Medical University.

Missing observations in the structural equation models were handled using full-information maximum likelihood estimation. Attrition analyses used chi-square and independent-samples t tests to compare participants who completed the follow-up assessments with those who did not. No statistically significant between-group differences were found for sex, χ²(1) = 3.312, p > 0.05, age, t(1179) = 0.475, p > 0.05, or any baseline study variable. The groups were comparable for Time 1 benefit finding, t(1179) = -0.682, p > 0.05, cognitive reappraisal, t(1179) = 1.01, p > 0.05, expressive suppression, t(1179) = 0.35, p > 0.05, social connectedness, t(1179) = 0.04, p > 0.05, depressive symptoms, t(1179) = 0.32, p > 0.05, and anxiety symptoms, t(1179) = 0.37, p > 0.05. Taken together, these results supported treating the missing observations as plausibly missing at random.

### 2.2 Measures

#### 2.2.1 Benefit finding

Benefit finding was assessed with the Chinese version of the Benefit Finding Scale (BFS-C). Antoni et al. (40) originally developed the scale to assess perceived positive changes among women undergoing diagnosis and treatment for breast cancer. Wang et al. (41) translated and culturally adapted the measure for use in China, and the Chinese version has subsequently been used in Chinese samples. The scale contains 17 items assessing personal and interpersonal benefits, including greater psychological strength, better preparation for future difficulties, and stronger family relationships. For the present study, the introductory instruction was adapted so that participants considered positive changes associated with the COVID-19 pandemic and rated each benefit as experienced during the previous week. Responses ranged from 1, indicating no perceived change, to 5, indicating a considerable amount of change. Item scores were summed, with higher total scores indicating greater benefit finding. Although the Chinese scale was initially evaluated in women with breast cancer, previous research reported good psychometric properties, including an internal consistency coefficient of 0.96 and a test-retest coefficient above 0.70 (41). In the present study, Cronbach’s alpha was 0.96 at Time 1, 0.96 at Time 2, and 0.90 at Time 3.

#### 2.2.2 Emotion regulation strategies

Habitual emotion regulation was assessed with the Emotion Regulation Questionnaire (ERQ) developed by Gross and John (27). This 10-item self-report measure contains two subscales. Six items assess cognitive reappraisal, or the tendency to change an emotional response by changing the interpretation of a situation. Four items assess expressive suppression, or the inhibition of emotional expression. Items are rated on a 7-point Likert scale ranging from 1, strongly disagree, to 7, strongly agree. Higher subscale scores indicate a greater tendency to use the corresponding strategy. Previous studies have shown good reliability and validity for the Chinese version among university students (42). In the present study at Time 1, Cronbach’s alpha was 0.92 for cognitive reappraisal and 0.76 for expressive suppression.

#### 2.2.3 Social connectedness

Perceived interpersonal connectedness was assessed with the Revised Social Connectedness Scale (SCS-R) developed by Lee, Draper, and Lee (43). This unidimensional 20-item measure assesses closeness, belonging, and interpersonal bonding. Items include statements about feeling close to other people and, in reverse-scored form, feeling that one does not truly belong even among familiar people. Participants responded on a 6-point Likert scale ranging from 1, strongly disagree, to 6, strongly agree. Negatively worded items were reverse scored before the total score was calculated, with higher total scores indicating greater subjective social connectedness. Previous research has supported the psychometric properties of the Chinese version (44). In the present sample, Cronbach’s alpha was 0.91 at Time 1, 0.92 at Time 2, and 0.93 at Time 3.

#### 2.2.4 Depression

Depressive symptoms were assessed with the Center for Epidemiologic Studies Depression Scale (CES-D) developed by Radloff (45) for use in community populations. The 20-item self-report measure assesses depressive symptoms across domains that include depressed mood, guilt or self-blame, helplessness and hopelessness, psychomotor agitation or retardation, appetite and weight changes, and sleep changes. Participants reported how frequently each symptom had occurred during the previous 7 days. Responses ranged from 0, hardly ever or not at all, to 3, most or all of the time. Item scores were summed to obtain a total score. Research in Chinese samples has supported the reliability and validity of the Chinese version (46). In the present study, Cronbach’s alpha was 0.89 at each of the three waves.

#### 2.2.5 Anxiety

Anxiety symptoms were assessed with the Beck Anxiety Inventory (BAI) developed by Beck, Epstein, Brown, and Steer (47). This 21-item self-report measure assesses the severity of common anxiety symptoms such as nervousness, fear of losing control, and expectation of a catastrophe. Participants rated how much they had been bothered by each symptom during the previous week on a 4-point scale ranging from 0, not at all, to 3, very much so and hardly able to stand it. Item scores were summed, with higher total scores indicating greater symptom severity. Previous research has supported the psychometric properties of the Chinese version among Chinese university students (48). In the present sample, Cronbach’s alpha was 0.96 at Time 1, 0.96 at Time 2, and 0.97 at Time 3.

### 2.3 Statistical analysis

SPSS version 22.0 was used to calculate descriptive statistics, pairwise correlations, and attrition analyses (chi-square tests and independent-samples t tests). Longitudinal trajectories of benefit finding, social connectedness, depressive symptoms, and anxiety symptoms were then analyzed with latent growth curve models in Mplus version 8.3 (49). Missing observations were incorporated through full-information maximum likelihood (FIML) estimation. Under the missing-at-random (MAR) assumption, this approach generally yields less biased estimates than listwise deletion and is appropriate for longitudinal structural equation modeling (50).

Following the conventional linear latent growth specification described by Bollen and Curran (13), two latent growth factors were defined for each repeatedly measured construct. The intercept represented estimated status at Time 1, and the slope represented linear change across the observation period. Factor loadings from all repeated measures to the intercept were fixed at 1. Because the three assessments were equally spaced by approximately 6 months, slope loadings were fixed at 0, 1, and 2 for Time 1, Time 2, and Time 3, respectively.

Parallel-process latent growth curve modeling was the primary analytic strategy for examining associations among social connectedness, emotion regulation, benefit finding, depression, and anxiety. The analysis proceeded in four steps.

#### 2.3.1 Step 1: unconditional linear growth models

Unconditional linear growth models were estimated separately for social connectedness, benefit finding, depressive symptoms, and anxiety symptoms to characterize the average trajectory of each construct. These models estimated the mean intercept and slope as well as interindividual variability in both growth factors.

#### 2.3.2 Step 2: model of variables associated with benefit finding

A parallel-process growth model was used to examine variables associated with the benefit finding trajectory. Cognitive reappraisal and expressive suppression measured at Time 1 were entered as time-invariant covariates. The intercept and slope of social connectedness were entered as model inputs for the intercept and slope of benefit finding. This model assessed whether baseline emotion regulation, initial social connectedness, and change in social connectedness were associated with the starting level and longitudinal change in benefit finding.

#### 2.3.3 Step 3: models linking benefit finding with mental health symptoms

Two additional parallel-process growth models examined the longitudinal associations of benefit finding with depressive and anxiety symptoms. In the depression model, the benefit finding intercept and slope were specified as model inputs for the depression intercept and slope. The anxiety model used the corresponding specification for anxiety. These models tested whether between-person differences in initial benefit finding and change in benefit finding were associated with differences in initial symptom levels and symptom change.

#### 2.3.4 Step 4: indirect association models

Parallel-process growth models were then used to examine indirect associations involving social connectedness, benefit finding, and mental health symptoms. Depression and anxiety were modeled separately. Bias-corrected bootstrap confidence intervals based on 5,000 resamples were used to evaluate indirect effects. The theory-specified model paths are summarized in **Figure 2**.

**Figure 2:**
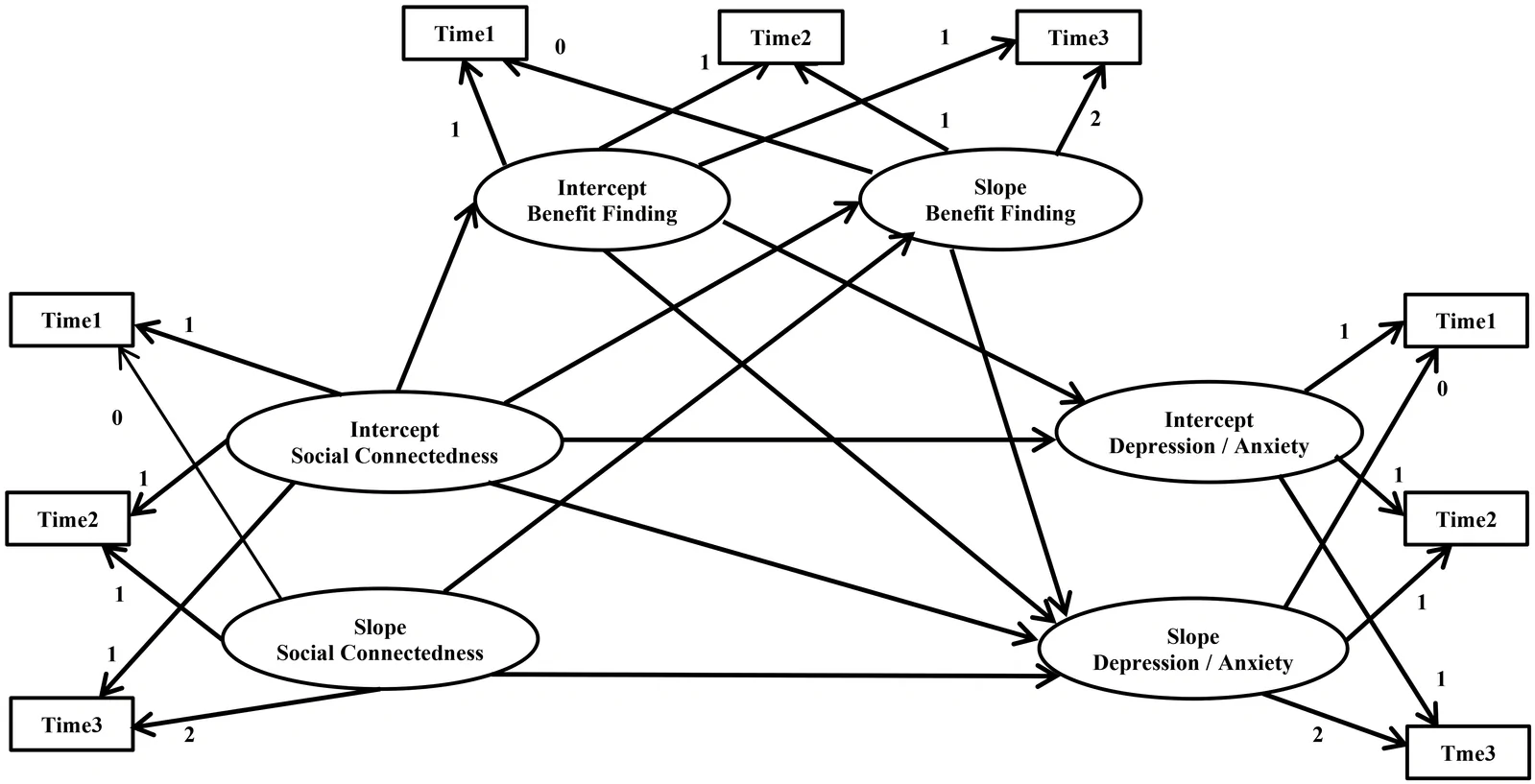
Parallel-process latent growth model used for the indirect association analyses. Depression and anxiety were estimated in separate models. Circles represent latent variables; rectangles represent observed measures. Single-headed arrows represent theory-specified statistical paths and do not establish causal direction.

These indirect effect analyses test whether the association between social connectedness and negative emotional symptoms can be explained in part by benefit finding, but they do not establish causal mediation or temporal precedence. The intercepts of different constructs were estimated from the same initial assessment, and the slopes reflect change over the same observation window. Reversing the paths among them would therefore not establish which construct came first. The parallel-process growth framework was retained because the primary research question concerns how initial levels, rates of change, and co-development are interrelated across constructs, rather than the direction of causal effects. The present findings should therefore be interpreted as longitudinal associations and hypothesis-generating evidence.

Model fit was evaluated with the comparative fit index, Tucker-Lewis index, root mean square error of approximation (RMSEA) with its 90% confidence interval, and standardized root mean square residual (51). Comparative fit index (CFI) and Tucker-Lewis index (TLI) values above 0.90 were considered acceptable and values above 0.95 excellent. Root mean square error of approximation values below 0.08 were considered good and values below 0.06 excellent.

## 3 Results

### 3.1 Descriptive analyses

Descriptive statistics and zero-order correlations for variables measured at each wave are shown in **Table 1**. Across the three assessments, greater cognitive reappraisal was associated with higher benefit finding, with correlations ranging from r = 0.123 to 0.154, all p < 0.01. Associations between expressive suppression and benefit finding were small or statistically nonsignificant. At each wave, greater social connectedness was associated with higher concurrent benefit finding, with correlations ranging from r = 0.210 to 0.348, all p < 0.01. Benefit finding was negatively correlated with depressive and anxiety symptoms at corresponding time points, with correlations ranging from r = -0.376 to -0.134, all p < 0.01. Repeated observations of the same construct generally showed moderate to high temporal correlations. For benefit finding, correlations were 0.449 between Time 1 and Time 2, 0.377 between Time 2 and Time 3, and 0.254 between Time 1 and Time 3, all p < 0.01. These coefficients indicate moderate temporal stability in benefit finding across the observation period.

**Table 1:** Descriptive statistics and bivariate correlations among study variables.

Variable	M	SD	1	2	3	4	5	6	7	8	9	10	11	12	13	14
1. Time 1 Cognitive Reappraisal	28.93	5.73	1													
2. Time 1 Expressive Suppression	16.89	4.08	.224**	1												
3. Time 1 Social Connectedness	74.28	5.55	.132**	-.030	1											
4. Time 2 Social Connectedness	75.30	5.45	.154**	-.030	.744**	1										
5. Time 3 Social Connectedness	76.17	5.94	.130**	-.091**	.501**	.664**	1									
6. Time 1 Benefit Finding	51.05	11.44	.123**	-.053	.348**	.293**	.210**	1								
7. Time 2 Benefit Finding	53.94	11.03	.123**	-.018	.241**	.312**	.198**	.449**	1							
8. Time 3 Benefit Finding	56.92	11.58	.046	-.079**	.104**	.204**	.281**	.254**	.377**	1						
9. Time 1 Depressive Symptoms	15.53	8.47	-.260**	.192**	-.345**	-.338**	-.302**	-.376**	-.328**	-.270**	1					
10. Time 2 Depressive Symptoms	14.55	8.40	-.206**	.131**	-.361**	-.385**	-.353**	-.313**	-.357**	-.317**	.723**	1				
11. Time 3 Depressive Symptoms	13.87	8.35	-.203**	.134**	-.321**	-.368**	-.395**	-.246**	-.299**	-.378**	.587**	.685**	1			
12. Time 1 Anxiety Symptoms	27.51	9.64	-.127**	.111**	-.225**	-.214**	-.179**	-.323**	-.271**	-.211**	.581**	.534**	.447**	1		
13. Time 2 Anxiety Symptoms	26.13	9.05	-.112**	.091**	-.176**	-.219**	-.188**	-.203**	-.293**	-.223**	.486**	.597**	.478**	.589**	1	
14. Time 3 Anxiety Symptoms	25.59	9.13	-.078**	.107**	-.158**	-.216**	-.246**	-.134**	-.222**	-.301**	.418**	.443**	.541**	.445**	.583**	1

Time 1, Time 2, and Time 3 occurred approximately 3, 9, and 15 months after restrictions were lifted. M, mean; SD, standard deviation. **p < 0.01, two-tailed.

### 3.2 Unconditional latent growth curve models

Univariate latent growth curve models were first estimated for social connectedness, benefit finding, depressive symptoms, and anxiety symptoms across the three waves. With three equally spaced assessments, a linear growth model was specified for each construct. The intercept represented estimated status at Time 1, approximately 3 months after restrictions ended, and the slope represented a constant linear rate of change across each 6-month interval. Intercept loadings were fixed at 1, and slope loadings were fixed at 0, 1, and 2 for the three waves (13).

**Table 2** summarizes fit statistics for the unconditional models. All four models fit the observed data well. Comparative fit index values ranged from 0.996 to 1.000, and root mean square error of approximation values ranged from 0.000 to 0.055. These results supported the use of linear trajectories over the 15-month post-restriction period.

**Table 2:** Fit indices for the unconditional latent growth curve models.

Variable	χ²	df	p	CFI	TLI	RMSEA [90% CI]	SRMR
Social Connectedness	0.604	1	0.437	1.000	1.001	0.000 [0.000, 0.071]	0.004
Benefit Finding	0.025	1	0.874	1.000	1.007	0.000 [0.000, 0.040]	0.001
Depressive Symptoms	0.976	1	0.323	1.000	1.000	0.000 [0.000, 0.077]	0.005
Anxiety Symptoms	4.554	1	0.033	0.996	0.989	0.055 [0.013, 0.111]	0.012

CFI, comparative fit index; TLI, Tucker-Lewis index; RMSEA, root mean square error of approximation; CI, confidence interval; SRMR, standardized root mean square residual.

**Table 3** presents the growth parameters. Social connectedness had a mean intercept of 74.288, p < 0.001, and a positive mean slope of 0.955 per 6-month interval, p < 0.001. Benefit finding also increased, with a mean intercept of 51.036, p < 0.001, and a mean slope of 2.934, p < 0.001. Depressive symptoms decreased from a mean intercept of 15.503, p < 0.001, with a mean slope of -0.840, p < 0.001. Anxiety symptoms also decreased, with a mean intercept of 27.376, p < 0.001, and a mean slope of -0.951, p < 0.001.

**Table 3:** Parameter estimates for the unconditional latent growth curve models.

Variable	Statistic	Intercept	Slope	Intercept variance	Slope variance	Intercept-slope covariance
Social Connectedness	Estimate	74.288***	0.955***	28.466***	5.485***	-5.987***
	SE	0.163	0.083	1.586	0.586	0.785
Benefit Finding	Estimate	51.036***	2.934***	79.799***	18.809***	-23.109***
	SE	0.328	0.206	7.348	3.493	4.304
Depressive Symptoms	Estimate	15.503***	-0.840***	61.311***	8.178***	-9.890***
	SE	0.248	0.112	3.629	1.315	1.682
Anxiety Symptoms	Estimate	27.376***	-0.951***	63.480***	10.552***	-12.162***
	SE	0.277	0.146	4.592	1.936	2.385

Social connectedness, benefit finding, depressive symptoms, and anxiety symptoms were modeled separately across three waves. SE, standard error. ***p < 0.001.

Significant interindividual variance was present in all intercept and slope factors, all p < 0.001, indicating significant variation in both initial levels and rates of change. Intercept-slope covariances were negative for all four constructs. For social connectedness, the covariance was -5.987 and the standardized coefficient was -0.479, p < 0.001. For benefit finding, the covariance was -23.109 and the standardized coefficient was -0.596, p < 0.001. For depressive symptoms, the covariance was -9.890 and the standardized coefficient was -0.442, p < 0.001. For anxiety symptoms, the covariance was -12.162 and the standardized coefficient was -0.470, p < 0.001. Thus, participants with higher initial social connectedness or benefit finding tended to show smaller subsequent increases, while participants with higher initial depressive or anxiety symptoms tended to show steeper subsequent decreases.

### 3.3 Variables associated with benefit finding

A parallel-process LGCM was used to examine variables associated with benefit finding trajectories. Cognitive reappraisal and expressive suppression measured at Time 1 were entered as time-invariant variables, and age and sex were included as covariates. The benefit finding intercept and slope were entered as model outputs for the corresponding social connectedness intercept and slope. The model showed adequate fit, χ²(25) = 85.512, p < 0.001, CFI = 0.975, TLI = 0.961, RMSEA = 0.046, 90% CI [0.035, 0.057], and SRMR = 0.034.

As shown in **Table 4**, the social connectedness intercept was positively associated with the benefit finding intercept, β = 0.427, 95% CI [0.362, 0.492], p < 0.001. Participants with higher social connectedness at Time 1 also reported higher benefit finding at Time 1. The slope factors were also positively associated. Greater increases in social connectedness coincided with steeper increases in benefit finding over the study period, β = 0.505, 95% CI [0.387, 0.623], p < 0.001.

**Table 4:** Standardized path coefficients for the model of variables associated with benefit finding.

Path	Estimate β	95% CI	p
Social connectedness intercept to benefit finding intercept	0.427	[0.362, 0.492]	< 0.001
Social connectedness slope to benefit finding slope	0.505	[0.387, 0.623]	< 0.001
Time 1 cognitive reappraisal to benefit finding intercept	0.128	[0.057, 0.199]	< 0.001
Time 1 expressive suppression to benefit finding intercept	-0.069	[-0.140, 0.002]	0.052
Sex to benefit finding intercept	-0.017	[-0.086, 0.052]	0.635
Age to benefit finding intercept	-0.005	[-0.074, 0.064]	0.878
Time 1 cognitive reappraisal to benefit finding slope	-0.112	[-0.206, -0.018]	0.020
Time 1 expressive suppression to benefit finding slope	0.029	[-0.065, 0.123]	0.542
Sex to benefit finding slope	0.006	[-0.084, 0.096]	0.892
Age to benefit finding slope	0.062	[-0.028, 0.152]	0.176
Benefit finding intercept with benefit finding slope	-0.579	[-0.697, -0.461]	< 0.001
Social connectedness intercept with social connectedness slope	-0.485	[-0.550, -0.420]	< 0.001
R² for benefit finding intercept	0.216		
R² for benefit finding slope	0.268		

β, standardized path coefficient; CI, bias-corrected bootstrap confidence interval; R², proportion of variance explained. Sex was coded 0 for female and 1 for male.

Cognitive reappraisal was positively associated with the benefit finding intercept, β = 0.128, 95% CI [0.057, 0.199], p < 0.001. Expressive suppression showed a small inverse association with the intercept that did not reach the conventional significance threshold, β = -0.069, 95% CI [-0.140, 0.002], p = 0.052. Cognitive reappraisal was negatively associated with the benefit finding slope, β = -0.112, 95% CI [-0.206, -0.018], p = 0.020, indicating that participants with higher baseline cognitive reappraisal tended to show a slower subsequent increase in benefit finding. Expressive suppression was not significantly associated with the benefit finding slope, β = 0.029, 95% CI [-0.065, 0.123], p = 0.542. Age and sex were not significantly associated with either benefit finding growth factor, all p > 0.05.

The covariance between the benefit finding intercept and slope was significantly negative, r = -0.579, p < 0.001. The corresponding covariance for social connectedness was also negative, r = -0.485, p < 0.001. Higher starting levels were therefore associated with more gradual subsequent increases. The variables in the model accounted for 21.6% of between-person variance in the benefit finding intercept and 26.8% of variance in its slope.

### 3.4 Associations of benefit finding with depression and anxiety

Two parallel-process growth models examined the associations of benefit finding with depressive and anxiety symptoms. These models tested whether individual differences in initial benefit finding and change in benefit finding were associated with differences in initial symptom levels and symptom change.

The depression model showed satisfactory fit, χ²(9) = 21.554, p = 0.010, CFI = 0.995, TLI = 0.991, RMSEA = 0.035, 90% CI [0.016, 0.054], and SRMR = 0.022. The anxiety model also fit adequately, χ²(9) = 26.420, p = 0.002, CFI = 0.990, TLI = 0.983, RMSEA = 0.041, 90% CI [0.023, 0.059], and SRMR = 0.023.

**Table 5** shows an inverse association between the benefit finding intercept and the depression intercept, β = -0.605, 95% CI [-0.666, -0.544], p < 0.001. Participants with higher benefit finding at Time 1 tended to report fewer depressive symptoms at Time 1. The slope factors were also inversely associated. A steeper increase in benefit finding coincided with a steeper decrease in depressive symptoms, β = -0.656, 95% CI [-0.829, -0.483], p < 0.001.

**Table 5:** Standardized path coefficients for the models linking benefit finding with mental health symptoms.

Path	Estimate β	95% CI	p
Benefit finding intercept to depressive symptom intercept	-0.605	[-0.666, -0.544]	< 0.001
Benefit finding slope to depressive symptom slope	-0.656	[-0.829, -0.483]	< 0.001
Benefit finding intercept to anxiety symptom intercept	-0.527	[-0.598, -0.456]	< 0.001
Benefit finding slope to anxiety symptom slope	-0.666	[-0.841, -0.491]	< 0.001
Depressive symptom intercept with depressive symptom slope	-0.432	[-0.556, -0.308]	< 0.001
Anxiety symptom intercept with anxiety symptom slope	-0.449	[-0.585, -0.313]	< 0.001
R² for depressive symptom intercept	0.366		
R² for depressive symptom slope	0.430		
R² for anxiety symptom intercept	0.278		
R² for anxiety symptom slope	0.443		

β, standardized path coefficient; CI, bias-corrected bootstrap confidence interval; R², proportion of variance explained.

A similar pattern was observed for anxiety. The benefit finding intercept was inversely associated with the anxiety intercept, β = -0.527, 95% CI [-0.598, -0.456], p < 0.001. Thus, higher benefit finding at Time 1 was associated with lower anxiety symptoms at Time 1. The slope factors were also inversely associated, β = -0.666, 95% CI [-0.841, -0.491], p < 0.001. Participants who showed greater increases in benefit finding tended to show steeper decreases in anxiety symptoms over time.

The intercept and slope of depressive symptoms covaried negatively, r = -0.432, p < 0.001, as did the intercept and slope of anxiety symptoms, r = -0.449, p < 0.001. Participants with higher initial symptom levels therefore tended to show faster decreases over time. In the depression model, benefit finding accounted for 36.6% of variance in the symptom intercept and 43.0% of variance in the symptom slope. The corresponding proportions in the anxiety model were 27.8% and 44.3%.

### 3.5 Indirect association analyses

Sex, age at Time 1, and baseline expressive suppression were not significantly associated with either benefit finding growth factor in the preceding model and were therefore omitted from the indirect association models. Cognitive reappraisal at Time 1 was retained as the time-invariant covariate because it was associated with the benefit finding growth factors and could also be associated with depressive or anxiety symptoms.

Two additional parallel-process growth models examined indirect associations linking social connectedness, benefit finding, and negative emotional symptoms. Depression and anxiety were modeled separately. In each model, the social connectedness intercept and slope were specified as model inputs, the corresponding benefit finding factors as intervening variables, and the depression or anxiety growth factors as model outputs. Baseline cognitive reappraisal was included as a covariate. These labels describe the statistical specification of the models and do not imply a demonstrated causal or temporal sequence.

In the unconstrained models, the residual variance of the negative emotion slope factor was estimated imprecisely. The estimate was 1.408 with an SE of 5.483 and p = 0.797 in the depression model and 1.340 with an SE of 8.284 and p = 0.872 in the anxiety model. The standard errors were several times larger than the estimates, and Mplus issued a nonpositive definite latent covariance matrix warning involving the slope factor. This pattern indicated an unstable boundary solution rather than reliable residual slope heterogeneity. Following established recommendations for improper or near-boundary variance estimates in structural equation models, the residual slope variance was constrained to a small positive value to stabilize estimation (52, 53). A value of 0.01 was selected for the primary models because it is close to the boundary while remaining positive. Sensitivity analyses then evaluated whether the substantive results changed when the variance was left unconstrained or fixed at 0.001, 0.01, or 0.10.

With the residual slope variance fixed at 0.01, the depression mediation model showed acceptable fit, χ²(29) = 122.035, p < 0.001, CFI = 0.980, TLI = 0.968, RMSEA = 0.053, 90% CI [0.043, 0.063], and SRMR = 0.052. The anxiety model also showed acceptable fit, χ²(29) = 114.184, p < 0.001, CFI = 0.977, TLI = 0.964, RMSEA = 0.050, 90% CI [0.041, 0.060], and SRMR = 0.049.

Three indirect pathways were evaluated in each model, as shown in **Table 6**. Statistical uncertainty was assessed with bias-corrected bootstrap confidence intervals based on 5,000 resamples. Because indirect effects commonly have asymmetric sampling distributions, the bias-corrected bootstrap confidence interval was treated as the primary inferential criterion, with the conventional normal-approximation p value reported as supplementary information (54, 55).

**Table 6:** Bootstrap indirect effects for the depression and anxiety models.

Indirect path	Effect	95% bias-corrected CI	Normal-approximation p
Depression model			
Social connectedness intercept through benefit finding intercept to depressive symptom intercept	-0.448	[-0.589, -0.333]	< 0.001
Social connectedness intercept through benefit finding slope to depressive symptom slope	0.204	[-0.021, 0.539]	0.113
Social connectedness slope through benefit finding slope to depressive symptom slope	-1.439	[-3.149, -0.680]	0.022
Anxiety model			
Social connectedness intercept through benefit finding intercept to anxiety symptom intercept	-0.477	[-0.646, -0.339]	< 0.001
Social connectedness intercept through benefit finding slope to anxiety symptom slope	0.230	[-0.054, 0.995]	0.191
Social connectedness slope through benefit finding slope to anxiety symptom slope	-1.805	[-5.905, -0.481]	0.064

Confidence intervals are based on 5,000 bias-corrected bootstrap resamples and are the primary inferential criterion for indirect effects. The normal-approximation p value is also reported. For the anxiety slope-level indirect effect, the bootstrap confidence interval excludes zero, whereas the normal-approximation p value is 0.064.

For depression, the indirect association linking the social connectedness intercept with the depression intercept through the benefit finding intercept was statistically supported, indirect effect = -0.448, 95% bias-corrected CI [-0.589, -0.333], p < 0.001. The slope-level indirect association was also supported. Greater increases in social connectedness coincided with greater increases in benefit finding, which in turn coincided with steeper decreases in depressive symptoms, indirect effect = -1.439, 95% bias-corrected CI [-3.149, -0.680], p = 0.022. The cross-domain indirect association from the social connectedness intercept to the depression slope through the benefit finding slope was not supported, indirect effect = 0.204, 95% bias-corrected CI [-0.021, 0.539], p = 0.113.

For anxiety, the indirect association linking the social connectedness intercept with the anxiety intercept through the benefit finding intercept was statistically supported, indirect effect = -0.477, 95% bias-corrected CI [-0.646, -0.339], p < 0.001. The slope-level indirect association was also supported, indirect effect = -1.805, 95% bias-corrected CI [-5.905, -0.481]. The corresponding normal-approximation p value was 0.064. Because the normal-theory test assumes a symmetric sampling distribution whereas bootstrap intervals are based on the empirical resampling distribution, and because methodological work generally recommends bootstrap confidence intervals for indirect effects when the sampling distribution is asymmetric (54, 55), the bootstrap confidence interval was treated as the primary inferential criterion. The cross-domain indirect association from the social connectedness intercept to the anxiety slope through the benefit finding slope was not supported, indirect effect = 0.230, 95% bias-corrected CI [-0.054, 0.995], p = 0.191.

Sensitivity analyses compared the unconstrained specification with models in which the residual negative emotion slope variance was fixed at 0.001, 0.01, or 0.10. Model fit was nearly identical across the three fixed values and was also very similar to the unconstrained models. Indirect effect estimates were stable in direction and magnitude across the fixed specifications. The bias-corrected confidence intervals were wider in the unconstrained models, especially for pathways involving the slope factor, but the substantive conclusions based on the bootstrap intervals were unchanged across the fixed values. These results indicate that the choice of 0.01 did not drive the main conclusions, although the need to constrain the variance remains a model limitation. Full results are reported in **Supplementary Table 1** through **4**.

## 4 Discussion

Consistent with H1a and H1b, benefit finding increased linearly across the 15-month post-restriction period, with significant interindividual variation in both initial levels and rates of change. Social connectedness also increased, whereas depressive and anxiety symptoms decreased. This phenomenon can be understood within the meaning-making model because renewed participation in classes, residential life, and campus activities may have provided repeated opportunities for students to reinterpret pandemic experiences and rebuild coherence (8). Recent studies of Chinese university students have likewise documented continuing mental health concerns and changes in psychological adjustment after restrictions were lifted (4, 56, 57). These studies primarily examined symptom trajectories. The trajectory of benefit finding itself, and its associations with psychosocial resources, have received less attention.

The trajectory of benefit finding observed here can also be compared with earlier longitudinal work on growth after collective adversity. Yan et al. (18), for example, identified several posttraumatic growth trajectories among Chinese healthcare workers during the acute phase of the pandemic, with the largest subgroup showing an increasing pattern. Whereas Yan et al. focused on the acute phase, the present study documented a continued increase during a later recovery period. This pattern differs from the rise followed by decline reported after the September 11 attacks (16). The COVID-19 pandemic unfolded over several years with successive phases of restriction and reopening, whereas the September 11 attacks were a single event concentrated within a single day. This difference in duration and recovery timeline may account for the contrasting trajectories. Within the meaning-making model, prolonged and phased adversity may allow more extended and iterative meaning-making than an acute, time-limited event (8). The sustained increase observed in the present study therefore suggests that benefit finding may continue to develop well beyond the initial crisis period.

At the individual level, cognitive reappraisal was positively associated with the benefit finding intercept but negatively associated with its slope, whereas expressive suppression was not significantly associated with either growth factor. According to the meaning-making model, the positive intercept association is understandable because cognitive reappraisal involves changing how an event is interpreted and therefore overlaps conceptually with meaning-related processing (8). Recent studies also link cognitive reappraisal with posttraumatic growth in pandemic contexts (58, 59). The negative slope association should be interpreted cautiously. One possibility is that students who already used reappraisal more frequently had higher initial benefit finding and therefore less room for further increase. Other unmeasured processes could also contribute to this pattern. Expressive suppression, by contrast, changes emotional expression without necessarily changing the meaning assigned to an experience (27), which is consistent with its weak associations in the present data. These findings identify cognitive reappraisal as a plausible target for future intervention research, although intervention studies are needed to test whether reappraisal training would increase benefit finding.

Social connectedness showed relatively strong associations with benefit finding in the present models. The social connectedness intercept was positively associated with the benefit finding intercept, β = 0.427, and the social connectedness slope was positively associated with the benefit finding slope, β = 0.505. These coefficients were larger in magnitude than the corresponding associations involving cognitive reappraisal. Within the meaning-making model, social connectedness represents an external resource that may facilitate the reinterpretation of stressful experiences (8). This pattern aligns with the thriving through relationships model (32) and with the resource accumulation logic of conservation of resources theory (34), although the present study did not directly test resource gain cycles. Consistent with this possibility, a longitudinal study of Chinese students documented changes in perceived social support after restrictions were eased (60). The post-restriction period may have been especially relevant because students regained opportunities for face-to-face interaction, classes, residential life, and campus activities. Through these everyday encounters, students could share their stresses and experiences, receive timely support, and strengthen friendships, which may have supported benefit finding. This relatively strong association may reflect relational orientations that are prominent in Chinese cultural contexts (61). The specified path direction was based on this theoretical and contextual rationale, but the data do not exclude an influence of benefit finding on later social connectedness or a reciprocal relationship between the two constructs.

Consistent with H4a and H4b, benefit finding was inversely associated with depressive and anxiety symptoms at both the intercept and slope levels. Students with higher benefit finding at the first assessment tended to report fewer symptoms at the same assessment, and students who showed greater increases in benefit finding tended to show steeper symptom declines. These associations are broadly consistent with the meaning-making model (8), which predicts that successful meaning-making is linked to reduced distress. For depression, restored meaning and perceived agency may be particularly relevant. reinterpreting the pandemic as an experience that clarified their priorities or strengthened their resilience may counter the hopelessness and negative self-appraisal that characterize depression. For anxiety, less threatening appraisals may be more directly relevant. recognizing that they have already coped with uncertain situations such as adjusting to new academic demands may provide evidence that future uncertainties are manageable. Longitudinal research among breast cancer survivors has linked benefit finding with subsequent reductions in depressive and anxiety symptoms (39), and Miao et al. (62) found that benefit finding was related to anxiety through hope, with their intervention study showing that daily benefit-finding writing increased benefit finding among Chinese college students. These findings support continued investigation of benefit finding as a potentially useful psychological resource, although experimental or intervention designs are needed to test whether benefit finding directly contributes to symptom reduction.

The indirect association models integrate the social connectedness, benefit finding, and symptom trajectories into a single longitudinal chain: social connectedness was associated with benefit finding, which in turn was associated with lower depressive and anxiety symptoms. This chain was evident at both the intercept and slope levels. This integrated pattern has practical implications for campus mental health services during the post-pandemic recovery period. Because social connectedness and benefit finding appeared to develop together, interventions that combine both—such as peer support groups or structured group reflection on pandemic experiences—may support both resources simultaneously, rather than targeting either in isolation. The results support an indirect association pattern involving benefit finding, but they do not establish a causal mediating mechanism. Reverse pathways, reciprocal influences, and unmeasured common causes remain possible explanations.

The findings suggest several directions for future research. First, subsequent experimental or intervention studies could examine whether enhancing social connectedness or facilitating benefit finding directly alleviates symptoms of depression and anxiety, further clarifying the underlying mechanisms among these variables. Second, future research could explore whether the common developmental patterns observed in this study can be generalized to other psychological resources (such as psychological resilience or self-efficacy). However, that the current observational analysis is insufficient to confirm that changing any of these variables alone would reduce levels of depression or anxiety.

### 4.1 Limitations and future directions

This study has several limitations. First, all constructs were assessed with self-report measures, which may introduce shared method variance and socially desirable responding. Future research could incorporate objective, clinician-rated, behavioral, or other multi-source assessments. Second, the sample included students from two Chinese universities, which limits generalizability to other educational and cultural contexts. Cross-cultural studies are needed to examine whether the strong association with social connectedness varies with relational or collectivist orientations. Third, only three time points were available, so the models could estimate linear change but not more complex nonlinear trajectories. More frequent assessments could identify periods of acceleration, stabilization, or decline. Fourth, other protective resources such as resilience and self-efficacy and risk processes such as rumination were not assessed. Future work could examine a broader set of individual and contextual factors within a multilevel framework.

The parallel-process latent growth curve model does not establish causal direction or a definitive temporal sequence among the growth factors. We therefore did not treat the theory-specified path direction as an empirical test of temporal precedence. Future studies could use random-intercept cross-lagged panel models with additional measurement occasions, intensive longitudinal designs, or experimental and intervention approaches to examine reciprocal and temporally ordered processes more directly (13). The indirect effect models in the present study represent statistical mediation rather than causal mediation. Finally, the residual slope variance for negative emotional symptoms had to be constrained to 0.01 in the indirect association models. Sensitivity analyses showed that the main indirect effect estimates and model fit were stable across several small fixed values, but the boundary problem may still affect slope-related precision. Replication with additional waves, larger samples, or alternative model specifications is warranted.

## 5 Conclusion

This study examined the development of benefit finding and its longitudinal associations with emotion regulation, social connectedness, depression, and anxiety among Chinese college students during post-pandemic recovery. Benefit finding and social connectedness increased linearly, while depressive and anxiety symptoms decreased. Cognitive reappraisal was associated with higher initial benefit finding but a slower subsequent increase, whereas expressive suppression was not significantly associated with either the intercept or the slope of benefit finding. Social connectedness showed stronger associations with benefit finding trajectories than the emotion regulation variables. Higher initial benefit finding and greater increases in benefit finding were associated with lower initial depressive and anxiety symptoms and steeper symptom declines. Indirect association patterns involving benefit finding were also observed between social connectedness and both symptom domains at the intercept and slope levels. These findings extend knowledge of psychological adjustment after a large-scale public health crisis while leaving the direction of the observed associations open for further testing. Relational and cognitive resources identified here warrant further investigation in culturally responsive campus mental health research.

## Data Availability

The raw data supporting the conclusions of this article will be made available by the authors, without undue reservation.
