# Supplementary material for: Longitudinal trajectories of benefit finding and its associations with emotion regulation, social connectedness, depression, and anxiety among Chinese college students following the removal of COVID-19 restrictions. a multivariate latent growth curve analysis
Source: Front Psychiatry. 2026 Oct 29;17:1940327. doi: 10.3389/fpsyt.2026.1940327 (PMC13755249; doi:10.3389/fpsyt.2026.1940327)
Supplement: Supplementary file 1 [file Table1.docx]

Supplementary Material

Sensitivity Analysis for the Slope Variance Constraint

In the main indirect association models, the residual variance of the negative emotion slope factor was fixed at 0.01 after the unconstrained models produced highly imprecise estimates and a nonpositive definite latent covariance matrix warning involving the slope factor. The value 0.01 was selected as a small positive value close to the boundary. The sensitivity analysis examined whether model fit and the indirect effect estimates remained stable when the variance was left unconstrained or fixed at 0.10, 0.01, or 0.001. Results are presented in Supplementary Tables 1 through 4.

**Supplementary Table 1. Model fit indices for the depression indirect association model across four conditions**

| **Residual slope variance** | **χ²** | **df** | **p** | **CFI** | **TLI** | **RMSEA [90% CI]** | **SRMR** |
| --- | --- | --- | --- | --- | --- | --- | --- |
| Unconstrained | 121.899 | 28 | < 0.001 | 0.979 | 0.967 | 0.054 [0.044, 0.064] | 0.052 |
| 0.10 | 122.021 | 29 | < 0.001 | 0.980 | 0.968 | 0.053 [0.043, 0.063] | 0.052 |
| 0.01 | 122.035 | 29 | < 0.001 | 0.980 | 0.968 | 0.053 [0.043, 0.063] | 0.052 |
| 0.001 | 122.036 | 29 | < 0.001 | 0.980 | 0.968 | 0.053 [0.043, 0.063] | 0.052 |

Note: Residual slope variance refers to the residual variance of the negative emotional symptom slope factor. The value 0.01 was used in the main manuscript. χ² = chi-square; df = degrees of freedom; CFI = comparative fit index; TLI = Tucker-Lewis index; RMSEA = root mean square error of approximation; CI = confidence interval; SRMR = standardized root mean square residual.

**Supplementary Table 2. Indirect effects for the depression model across four conditions**

| **Indirect effect** | **Unconstrained** | **0.10** | **0.01** | **0.001** |
| --- | --- | --- | --- | --- |
| Social connectedness intercept via benefit finding intercept to depressive symptom intercept | -0.451 [-0.595, -0.336], p < 0.001 | -0.448 [-0.589, -0.333], p < 0.001 | -0.448 [-0.589, -0.333], p < 0.001 | -0.448 [-0.589, -0.333], p < 0.001 |
| Social connectedness intercept via benefit finding slope to depressive symptom slope | 0.163 [-0.029, 1.149], p = 0.430 | 0.201 [-0.021, 0.533], p = 0.113 | 0.204 [-0.021, 0.539], p = 0.113 | 0.205 [-0.021, 0.539], p = 0.113 |
| Social connectedness slope via benefit finding slope to depressive symptom slope | -1.155 [-6.424, -0.458], p = 0.327 | -1.420 [-3.111, -0.666], p = 0.022 | -1.439 [-3.149, -0.680], p = 0.022 | -1.441 [-3.154, -0.682], p = 0.022 |

Note: Values are indirect effect estimates with 95% bias-corrected bootstrap confidence intervals in brackets and normal-approximation p values. The bootstrap confidence interval is the primary inferential criterion for indirect effects. Under the unconstrained condition, confidence intervals were wider for all indirect effects, with the largest increase for paths involving the slope factor.

**Supplementary Table 3. Model fit indices for the anxiety indirect association model across four conditions**

| **Residual slope variance** | **χ²** | **df** | **p** | **CFI** | **TLI** | **RMSEA [90% CI]** | **SRMR** |
| --- | --- | --- | --- | --- | --- | --- | --- |
| Unconstrained | 114.145 | 28 | < 0.001 | 0.976 | 0.962 | 0.052 [0.042, 0.062] | 0.049 |
| 0.10 | 114.179 | 29 | < 0.001 | 0.977 | 0.964 | 0.050 [0.041, 0.060] | 0.049 |
| 0.01 | 114.184 | 29 | < 0.001 | 0.977 | 0.964 | 0.050 [0.041, 0.060] | 0.049 |
| 0.001 | 114.184 | 29 | < 0.001 | 0.977 | 0.964 | 0.050 [0.041, 0.060] | 0.049 |

Note: Residual slope variance refers to the residual variance of the negative emotional symptom slope factor. The value 0.01 was used in the main manuscript. χ² = chi-square; df = degrees of freedom; CFI = comparative fit index; TLI = Tucker-Lewis index; RMSEA = root mean square error of approximation; CI = confidence interval; SRMR = standardized root mean square residual.

**Supplementary Table 4. Indirect effects for the anxiety model across four conditions**

| **Indirect effect** | **Unconstrained** | **0.10** | **0.01** | **0.001** |
| --- | --- | --- | --- | --- |
| Social connectedness intercept via benefit finding intercept to anxiety symptom intercept | -0.478 [-0.717, -0.306], p < 0.001 | -0.477 [-0.646, -0.339], p < 0.001 | -0.477 [-0.646, -0.339], p < 0.001 | -0.477 [-0.646, -0.339], p < 0.001 |
| Social connectedness intercept via benefit finding slope to anxiety symptom slope | 0.199 [0.011, 2.076], p = 0.374 | 0.228 [-0.053, 0.983], p = 0.191 | 0.230 [-0.054, 0.995], p = 0.191 | 0.230 [-0.053, 0.996], p = 0.191 |
| Social connectedness slope via benefit finding slope to anxiety symptom slope | -1.563 [-9.523, -0.660], p = 0.284 | -1.788 [-5.826, -0.479], p = 0.064 | -1.805 [-5.905, -0.481], p = 0.064 | -1.807 [-5.913, -0.481], p = 0.064 |

Note: Values are indirect effect estimates with 95% bias-corrected bootstrap confidence intervals in brackets and normal-approximation p values. The bootstrap confidence interval is the primary inferential criterion for indirect effects. Under the unconstrained condition, confidence intervals were wider for all indirect effects, with the largest increase for paths involving the slope factor.
